# Identification of *Toxoplasma gondii* antigenic proteins using an *in vivo* approach and *in silico* investigation of their polymorphism

**DOI:** 10.1128/spectrum.02040-24

**Published:** 2025-03-26

**Authors:** J. Denis, C. Gommenginger, L. Beal, B. Cimon, A. S. Deleplancque, H. Fricker Hidalgo, C. L'Ollivier, L. Paris, H. Pelloux, C. Pomares, S. Houze, A. W. Pfaff, I. Villena, O. Villard

**Affiliations:** 1 Institut de Parasitologie et de Pathologie Tropicale, UR7292 Dynamique des interactions hôte pathogène, Fédération de Médecine Translationnelle, Université de Strasbourg 568434 , Strasbourg, Grand Est, France; 2 Laboratoire de Parasitologie et Mycologie Médicale, Hôpitaux Universitaires de Strasbourg, Strasbourg, France; 3 Centre National de Référence Toxoplasmose-Pôle sérologie, Hôpitaux Universitaires de Strasbourg, Strasbourg, France; 4 Laboratoire de Parasitologie-Mycologie, CHU d'Angers 26966, Angers, Pays de la Loire, France; 5 Université d’Angers, IRF, SFR 4208 ICAT, Angers, Pays de la Loire, France; 6 CHU Lille, Département de Parasitologie-Mycologie, Université de Lille, Inserm, U995-LIRIC–Lille Inflammation Research International Center, Lille, France; 7 Laboratoire de Parasitologie et Mycologie Médicale, CHU de Grenoble-Alpes, Institute for Advanced Biosciences, INSERM U1209, CNRS UMR5309, CHU de Grenoble-Alpes, Grenoble, France; 8 IHU-Méditerranée Infection, Assistance Publique Hôpitaux de Marseille (AP-HM), Marseille, France; 9 IRD, AP-HM, SSA, VITROME, IHU Méditerranée, Université d’Aix Marseille, Marseille, France; 10 Laboratoire de Parasitologie, Hôpital Pitié-Salpêtrière, AP-HP Sorbonne Université, Paris, France; 11 Laboratoire de Parasitologie et Mycologie Médicale, CHU Nice, INSERM 1065, C3M, Université Côte d’Azur, Nice, France; 12 Laboratoire de Parasitologie et Mycologie Médicale, AP-HP, Hôpital Bichat–Claude Bernard, Paris, France; 13 Université Paris Cité, IRD 261, MERIT, Paris, France; 14 Laboratoire de Parasitologie et Mycologie Médicale, Centre National de Référence de la Toxoplasmose, CHU de Reims, Reims, France; 15 EA 7510, SFR CAP-SANTE, Université Reims Champagne Ardenne, Reims, France; Hebrew University of Jerusalem, Jerusalem, Israel

**Keywords:** *Toxoplasma*, antigenic protein, mass spectrometry, polymorphism, variant call analysis

## Abstract

*Toxoplasma gondii* is a pathogen characterized by a large variety of strains whose virulence and clinical severity are likely linked to their genotype. Currently, the strains are genotyped using restriction fragment length polymorphism, multilocus sequence typing, and microsatellite markers. This typing requires the strain’s DNA, which is difficult to obtain. A serotyping test could overcome the constraints of genotyping, the challenge being to identify type-specific proteins. We identified immunogenic *T. gondii* proteins from co-immunoprecipitations for three tachyzoite strains (strain FOU from Africa 1 type, ME49 from Type II, and VEG from Type III) with hyperimmune murine sera and conducted an *in silico* polymorphism search for the identified proteins. A variant calling analysis was conducted on the next-generation sequencing sequences of 117 *T. gondii* isolates with the objective of identifying mutations present in the genes encoding the antigenic proteins previously identified. A total of 727 immunogenic proteins were identified, including 16% dense granule protein (GRA), rhoptry protein (ROP/RON), and surface antigen protein (SAG). Genetic analysis revealed the presence of 36 single-nucleotide polymorphisms (SNPs) in over 70% of isolates belonging to the same type, while less than 30% of isolates belonging to the other types exhibited these polymorphisms. Of these, only 15 are located on coding DNA sequence regions, while four are located on genes encoding apicomplexan proteins: two SNPs on the ROP5 gene and two on the ROP7 gene. The results of this study indicate that a significant number of *T. gondii* immunogenic proteins can be identified using an *in vivo* approach. The *in silico* study identified SNPs that could be genotype-specific.

**IMPORTANCE:**

*Toxoplasma gondii* is a unique species that exhibits genotype diversity related to clinical virulence. Currently, genotyping is restricted, which limits epidemiological knowledge of the strains. To overcome this limitation, we aimed to develop serotyping tests. First, we used a murine *in vivo*, non-targeted experimental approach based on proteomics techniques through which we were able to identify a panel of more than 700 antigenic proteins from *T. gondii*. Then, we analyzed the polymorphism of these proteins using a whole-genome sequencing database containing the genomes of 117 genotyped strains. We showed that none of the 986 non-silent SNPs detected is specific to the strain type. The *in vivo* approach is the first that allowed the identification of such a large panel of antigenic proteins. Moreover, the polymorphism analysis, the first based on a large next-generation sequencing database, showed the limits that currently restrict the development of a serotyping technique.

## INTRODUCTION

*Toxoplasma gondii* (*T. gondii*) is a pathogen that infects many animals and for which humans are a parasitic dead-end. The *Toxoplasma* genus is represented by a single species, within which there is a diversity of strains depending on geographical distribution and virulence (1–4). There are several genetic classifications of these strains. The original one describes a clonal population with three lineages, namely, Types I, II, and III (2), and the so-called “atypical” strains, which display greater genetic diversity (1). Since then, the classifications have evolved by grouping the strains into six clades and 16 haplogroups based on restriction fragment length polymorphism, multilocus sequence typing (MLST), and microsatellite markers (MS) (5, 6).

Among these lineages, Types II and III are considered non-virulent and potentially cystogenic in mice and humans (7, 8). Type II strains are the most widespread, particularly in Europe (90%), Africa (54%), and North America (38%) (9). Type I and atypical strains are more virulent. They are notably responsible for severe pulmonary, cerebral, or ocular damage, which does not appear to be correlated with the host’s immune status. They are mainly isolated in South America, such as the Amazonian strains (83%), and in Asia (18% for Type I and 81% for atypical strains). Africa 1, detected for the first time in domestic cats, seems to be as virulent as Type I strains. It has been isolated in some cases of congenital toxoplasmosis (10–13) and severe toxoplasmosis in immunocompetent patients (14). Therefore, links appear between the strain’s genotype and its virulence in humans. Better identification of the strain types associated with severe clinical manifestations could improve the management of these cases. However, the precise link between the strain type and the pathophysiology of the infection is difficult to establish. Indeed, strain genotyping can only be performed with a sufficient amount of *T. gondii* DNA in the samples. This condition is rarely achieved in patients, as the infection is asymptomatic in most cases (15, 16). A serotyping technique that enables indirect strain typing by antibody specificity could be a tool of paramount importance for better describing *T. gondii* epidemiology and improving patient management.

The objective of serotyping is to identify peptide sequences that are specific to a particular antibody type. These peptides can be used to detect qualitative or quantitative differences in antibody populations that are associated with different strains of the infecting agent. The development of typing tests is based on *in silico* analysis of antigenic proteins whose polymorphism is type-specific. Subsequently, the type specificity of these proteins/peptides is subjected to experimental testing.

The most widely studied immunogenic proteins to date are those derived from apicomplexan organelles, such as dense granule proteins (GRA), microneme proteins (MIC), rhoptry (ROP/RON) proteins, and surface antigens (SAG) (17–28). A recent study investigated a wider panel of proteins by integrating *in silico* polymorphism analysis on proteins having a signal peptide (18). Indeed, secreted and surface proteins are the most likely to be immunogenic. Overall, approximately 70 *T. gondii* proteins have been analyzed for the development of serotyping tests. These include GRA6 and GRA7, the most extensively studied proteins, as well as GRA3, GRA4, and GRA5, SAG1, SAG2, SAG3, and SAG4, and ROP1, ROP5, ROP8, ROP16, ROP18, and ROP20 (17–28). To date, serotyping tests have been developed based on type-specific peptides obtained from these proteins.

The current serotyping techniques have been demonstrated to have limited performance. While they are capable of distinguishing Type II from non-Type II, the differentiation of other types is more unreliable. With the exception of certain proteins, peptide identification is most often based on studies of polymorphism in a small number of isolates for each genotype. This approach presents a challenge in determining with certainty that the observed polymorphism is type-specific. Conversely, the majority of studies compare the sequences of Type I, II, and III strains, with only a few including sequences of other types (e.g., Africa, Type 12) (18). It would, therefore, be of interest to study the polymorphism of several strain genotypes represented by a large number of individuals. A further limitation to the development of typing tests is the limited number of proteins that have been studied. These proteins are presumed to be antigenic, such as apicomplexan proteins. The number of *T. gondii* proteins that have been demonstrated to be antigenic is currently limited to approximately 50 (17–26, 28, 29).

Against this background, the aim of our work was to identify novel candidate targets for the future development of serotyping tests. To this end, we used an *in vivo* approach for the identification of *T. gondii* antigenic proteins. This involved co-immunoprecipitation of *T. gondii* lysate and infected mouse sera, followed by liquid chromatography–tandem mass spectrometry (LC–MS/MS) detection. Subsequently, an *in silico* analysis of the polymorphism present in the genes encoding these proteins was performed with the aim of identifying mutations on antigenic sequence whose frequency can be linked to genotype. To identify mutations of interest, we used a whole-genome sequencing database of 117 strains divided into four major and five minor genotypes.

## MATERIALS AND METHODS

### Identification of immunogenic proteins

#### Parasite and mice

The strains FOU (TgH00007, Africa 1), ME49 (TgA00001, Type II), and VEG (TgH00005, Type III) (CRB *Toxoplasma*) were grown as tachyzoites *in vitro* on VERO cells (ATCC CCL-81). Female Swiss Webster mice aged at least 6 weeks (RjORL:Swiss) (January) were used for infections

#### Hyperimmune murine sera

Mice were infected via intraperitoneal injections of tachyzoites derived from *in vitro* culture of the three strains. The infectious doses were 5,000 tachyzoites for FOU and 20,000 tachyzoites for Me49 and VEG. Mice were treated with sulfadiazine (60 mg/kg/d) on D1 post-infection. Re-infection occurred 4 weeks after primary infection. Mouse sera were collected 10 weeks after primary infection. The infections were performed in triplicate. The semi-quantitative determination of serum antibodies to *T. gondii* was performed via hemagglutination with the ELI.H.A TOXO Kit (Elitech), while the total IgG in sera was measured with the Procartaplex Mouse Antibody Isotyping Panel 7-plex Kit (Invitrogen) according to the manufacturer’s recommendations.

#### Co-IP

Co-IP reactions between pooled hyperimmune murine sera and *T. gondii* tachyzoite lysates were performed: homologous (hyperimmune murine serum vs. the strain that infected the mouse) and heterologous reactions (hyperimmune serum vs. the other two strains). Overall, three homologous and six heterologous Co-IP reactions were performed in triplicate using the Pierce Classic Magnetic IP/Co-IP Kit (Thermo Scientific) according to the manufacturer’s recommendations. In brief, 10^8^ tachyzoites from *in vitro* cultures were lysed, then 500 µg of antigen was brought into overnight contact with mouse sera at +4°C. The amount of serum was adjusted to reach 2 µg of total IgG. Total IgGs were assayed using the Procartaplex Mouse Antibody Isotyping Panel 7-plex Kit (Invitrogen). Elution was performed under reducing conditions.

#### Identification of immunogenic proteins

Proteins in the eluates were identified by LC–MS/MS on the Institut Pluridisciplinaire Hubert Curien platform (https://plateforme-psge.u-strasbg.fr/PLATEFORME_LSMBO_WEB/UK/) and by comparison with the ME49 reference strain via the ToxoDB and Mascot database (30, 31). The quality of the identification of each protein was assessed on the basis of coverage rates and the number of specific peptides used to identify them. Proteins were categorized using the Gene Ontology (GO) classification with PANTHER (v17.0). All proteins identified in Co-IP reactions were included in the analysis, regardless of the number of replicates in which they were detected. The mass spectrometry proteomics data have been deposited to the ProteomeXchange Consortium via the PRIDE partner repository with the data set identifiers PXD058695 and 10.6019/PXD058695.

#### *In silico* detection of antigenic protein polymorphism

**Fig 1:**
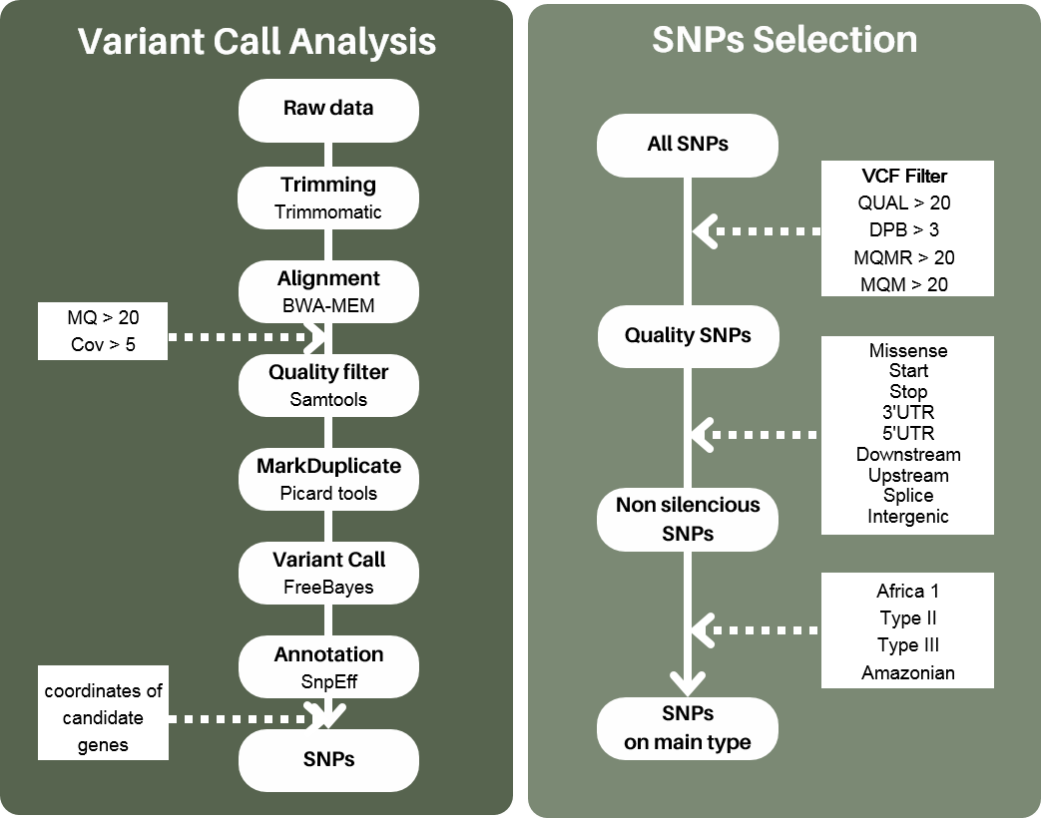
Study workflow. Variant calling analysis and SNP selection workflow. MQ: mapping quality. Cov: coverage. QUAL: quality. DPB: total read depth per bp at the locus. MQMR: mean mapping quality of observed reference alleles. MQM: mean mapping quality of observed alternate alleles.

An *in silico* polymorphism analysis of the antigenic proteins detected by Co-IP was conducted. The genetic data used in this study were derived from 117 *T. gondii* isolates, with 97 isolates from Africa 1 (*n* = 15), Type II (*n* = 48), Type III (*n* = 19), and Amazonian (*n* = 15) and 20 isolates from Caribbean (*n* = 7), Africa 3 (*n* = 4), Africa 4 (*n* = 3), Type I (*n* = 3), and Type 12 (*n* = 3) (Table 1) (32). The complete genomes obtained by next-generation sequencing (NGS) of the 117 isolates were downloaded from the ENA platform (https://www.ebi.ac.uk/ena/browser/home) (Supp 1.) (33). The sequences were then cleaned and aligned against the Me49 reference genome (GCA_000006565.2, 2018) via BWA-MEM (v0.7.17.2). The alignments were sorted with Samtools sort (v2.0.4), and duplicates were marked with MarkDuplicate (Picard tools, v2.18.2.3). Alignments with a mapping quality below 20 and coverage below 5 were excluded (MQ < 20; Cov < 5). To identify single-nucleotide polymorphisms (SNPs) in antigenic protein-coding genes, variant calling was performed using FreeBayes (v1.3.6). Identified variants were annotated using SNpEff (v4.3+T.) based on annotations from the Me49 reference genome (GCA_000006565.2, 2018). Annotated variant calls were merged, focusing on gene coordinates identified using bcftools merge (v1.15.1). Raw data were filtered in two steps using the VCF filter: first, SNP variants were selected, followed by variants meeting the criteria of QUAL > 20, DPB > 3, MQMR > 20, and MQR > 20 (Fig. 1). All analyses were performed on the Galaxy platform (https://usegalaxy.eu), and data were made available (32).

**TABLE 1:** Isolate distribution within genotypes^*a*^

Genotype name	No. of isolate
	(Human)
Africa 1	15 (5)
Type II	48 (4)
Type III	19 (1)
Amazonian	15 (3)
Autre :	20 (6)
Type I	3
Type 12	3 (2)
Africa 3	4
Africa 4	3
Caribbean	7 (4)

^*a*^ Type of *T. gondii* strains used in the polymorphism study. Strains were typed on 15 microsatellite markers (32).

### Polymorphism analysis

Filtered variants were then distinguished between their association with a non-silent protein polymorphism on coding sequences (CDS) (effects: missense variant; start lost; stop gained; stop lost; stop retained) and regulatory regions (effects: 3′ UTR; 5′ UTR; downstream variant; upstream variant; splice region; intergenic region) (Fig. 1). The distribution of these SNPs was then assessed by calculating the percentage of mutated isolates in total and on each type (10.6084/m9.figshare.27118695). The mutations of interest were selected from the 97 isolates distributed over the four types: Africa 1, II, III, and Amazonian, given the higher number of isolates for these types. SNPs were included if the mutation was present in more than 70% of isolates of a given type and less than 30% of isolates of the other three types. Additionally, the distribution of selected mutations in the 20 additional isolates of the Caribbean, Africa 3, Africa 4, Type I, and Type 12 types was analyzed.

## RESULTS

### Characterization of murine sera

All murine sera obtained were hemagglutination-positive, with anti-*T*. *gondii* Ab titers ranging from 1/80 to 1/2,560 and total IgG concentrations ranging from 6,926 to 1,383,136 ng/mL (Table S1). The sera from mice infected with the FOU strain exhibited the lowest anti-*T*. *gondii* Ab titers.

### Identification of antigenic protein

**Fig 2:**
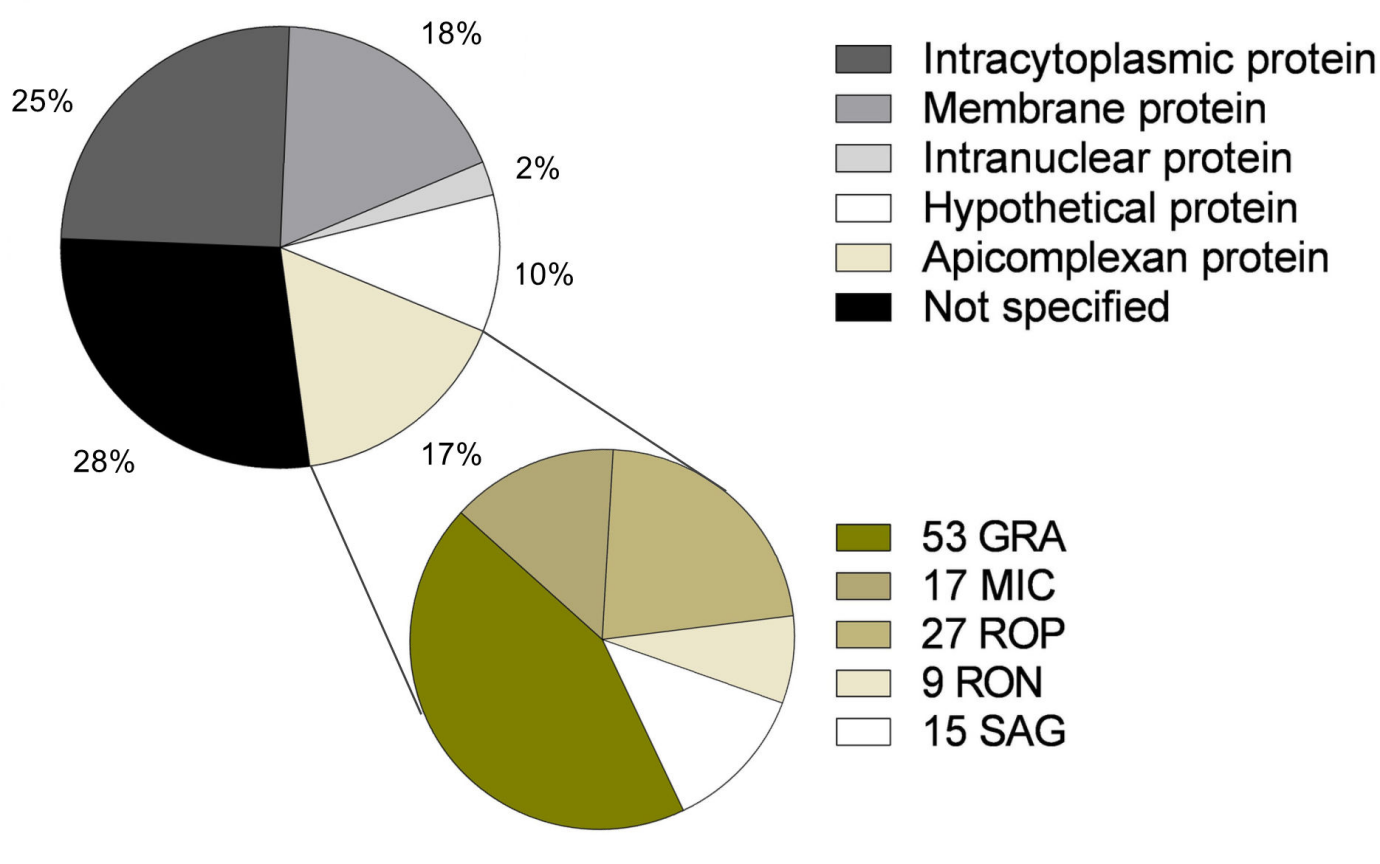
Distribution of the proteins identified by co-immunoprecipitation (Co-IP) coupled to mass spectrometry according to cell location/type.

Co-immunoprecipitation reactions coupled with LC–MS/MS enabled the detection of 727 proteins recognized by murine anti-*T*. *gondii* Ig. Of the 727 proteins identified, 25% (182/727) were found to be intracellular; 18% (131/727) were membrane proteins; 17% (121/727) were apicomplexan proteins; 10% (73/727) were hypothetical proteins; 2% (18/727) were intra-nuclear proteins; and 28% (202/727) were not referenced in the GO classification (Fig. 2).

**Fig 3:**
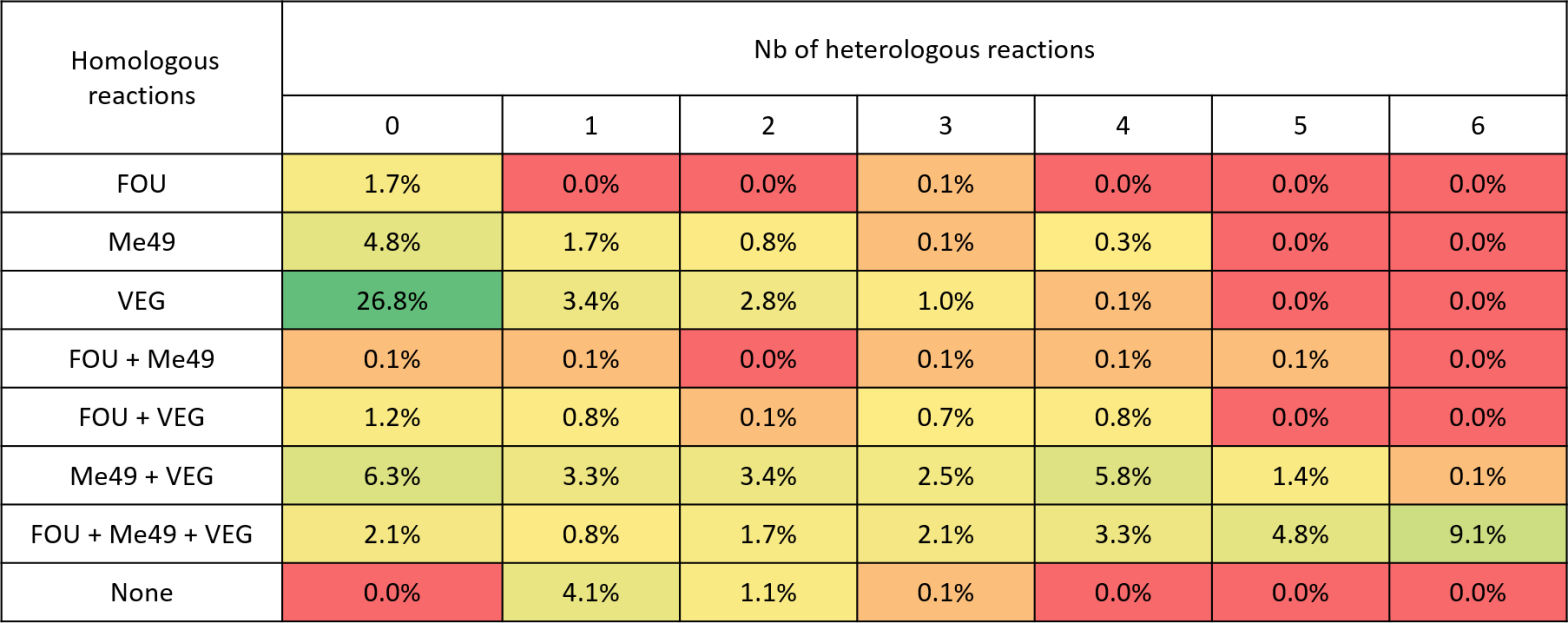
Distribution of proteins according to the reaction in which they were detected. Homologous (hyperimmune murine serum vs. the strain that infected the mouse) and heterologous reactions (hyperimmune serum vs. the other two strains) were performed. FOU: reaction between sera from mice infected by FOU strain and FOU lysate; Me49: reaction between sera from mice infected by Me49 strain and Me49 lysate; and VEG: reaction between sera from mice infected by VEG strain and VEG lysate. From red: no protein detected to green: >25% of the protein detected during the Co-IP reaction.

Of these 727 proteins, 43% (313/727) were detected only in homologous reactions, while 5% (39/727) were detected only in heterologous reactions (Fig. 3). The remaining 375 proteins were identified in at least one heterologous reaction. Of the apicomplexan proteins, 20% (24/121) were found in homologous reactions alone, with the remainder found in at least one of the heterologous reactions. These include GRA3, GRA4, GRA5, GRA6, and GRA7, as well as ROP1, ROP5, ROP8, ROP16, and ROP18.

Of the 727 proteins, 686 (94%) were described for the first time as being recognized by murine anti-*T*. *gondii* Ig. Of the 121 apicomplexan proteins identified in this study, 36 have previously been described as antigenic and studied in typing tests, whereas 83 of the proteins are described as recognized by anti-*T*. *gondii* Ig for the first time, including 40 GRA, 13 MIC, nine RON, 14 ROP, and seven SRS.

### Polymorphism description

**Fig 4:**
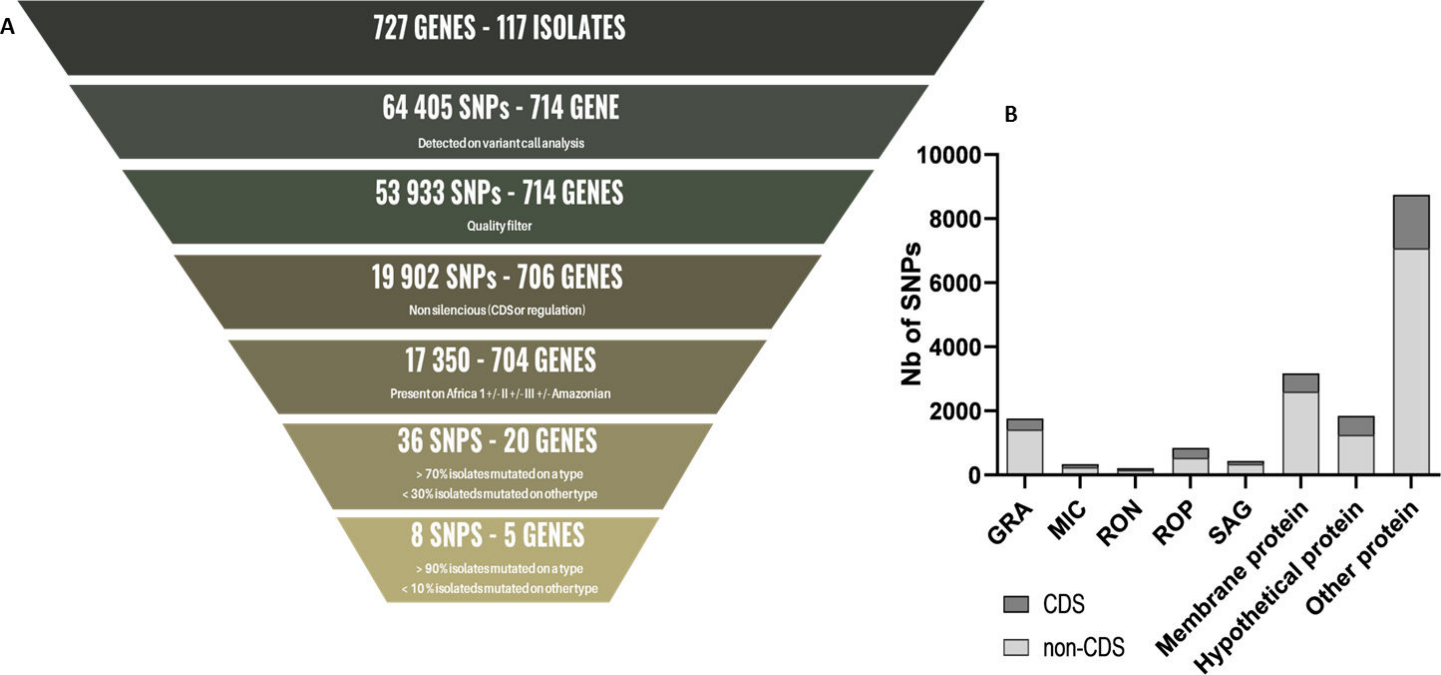
Description of SNPs detected by variant calling analysis performed on NGS genetic data from 117 *T. gondii* isolates. (A) Number of SNPs included. (B) Categorization of SNPs according to the protein encoded by the gene. CDS: SNPs present on a coding sequence. Non-CDS: SNPs present in a regulatory region.

A total of 53,933 SNPs with sufficient quality were identified through variant calling analysis of the 727 genes of interest. Of these, only 32% (17,350/53,933) are non-silent and located on at least one of the 97 isolates from the Africa 1, II, III, and Amazonian types (Fig. 4A). These SNPs are distributed over 704 of the 727 genes of interest: 17% (119/704) are apicomplexan protein genes; 10% (70/704) are genes encoding hypothetical proteins; 18% (128/704) are membrane protein genes; and 55% (387/704) are genes encoding other proteins (Fig. 4B). On average, 24 SNPs were observed per gene (median = 17, Q1 = 1, Q3 = 30). Of the 17,350 SNPs of interest, 3,697 are located on a CDS region and 13,653 on a regulatory region of the gene of interest. These SNPs are distributed over 684 and 470 genes, respectively.

**Fig 5:**
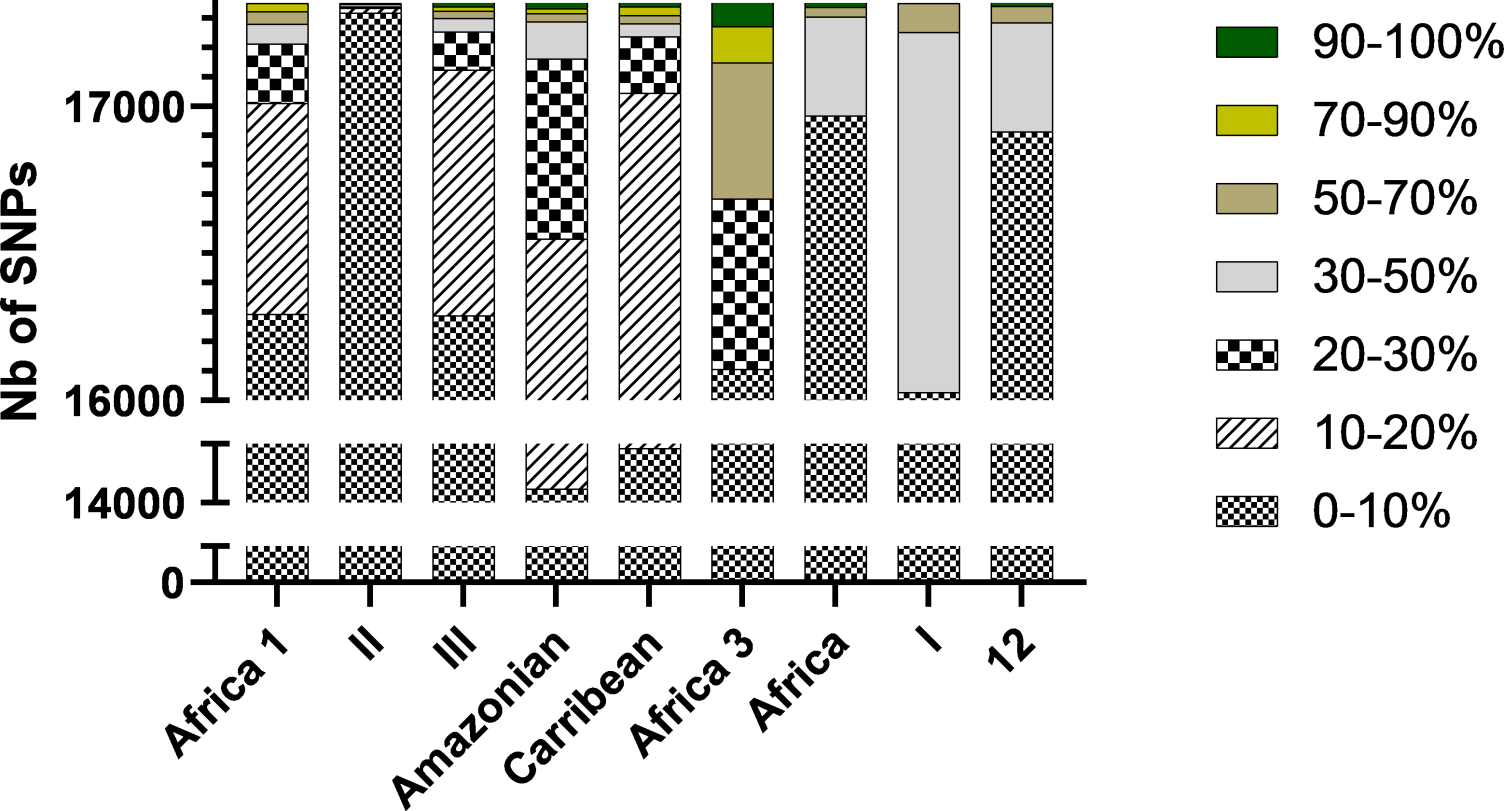
Total number of SNPs observed in each type. Within each type, there are percentages of strains with the same SNP.

For each of the 17,350 SNPs, the average number of mutated isolates is 1 (with a median of 1, a maximum of 52, a first quartile of 1, and a third quartile of 2). Type II isolates showed the greatest degree of homogeneity, with only 0.1% (2/17,351) of SNPs having a mutation rate greater than 30% across Type II isolates. In contrast, for the other types, the isolates showed a mutation rate greater than 30% (0.8% of the SNPs for Africa 1, 0.6% for III, and 1.1% for Amazonian) (Fig. 5). Furthermore, the proportion of mutated isolates per type is higher for SNPs present in the CDS with 1.5, 0.1, 1.1, and 2.2% for types Africa 1, II, III, and Amazonian, respectively.

### Distribution of SNPs on isolates

**Fig 6:**
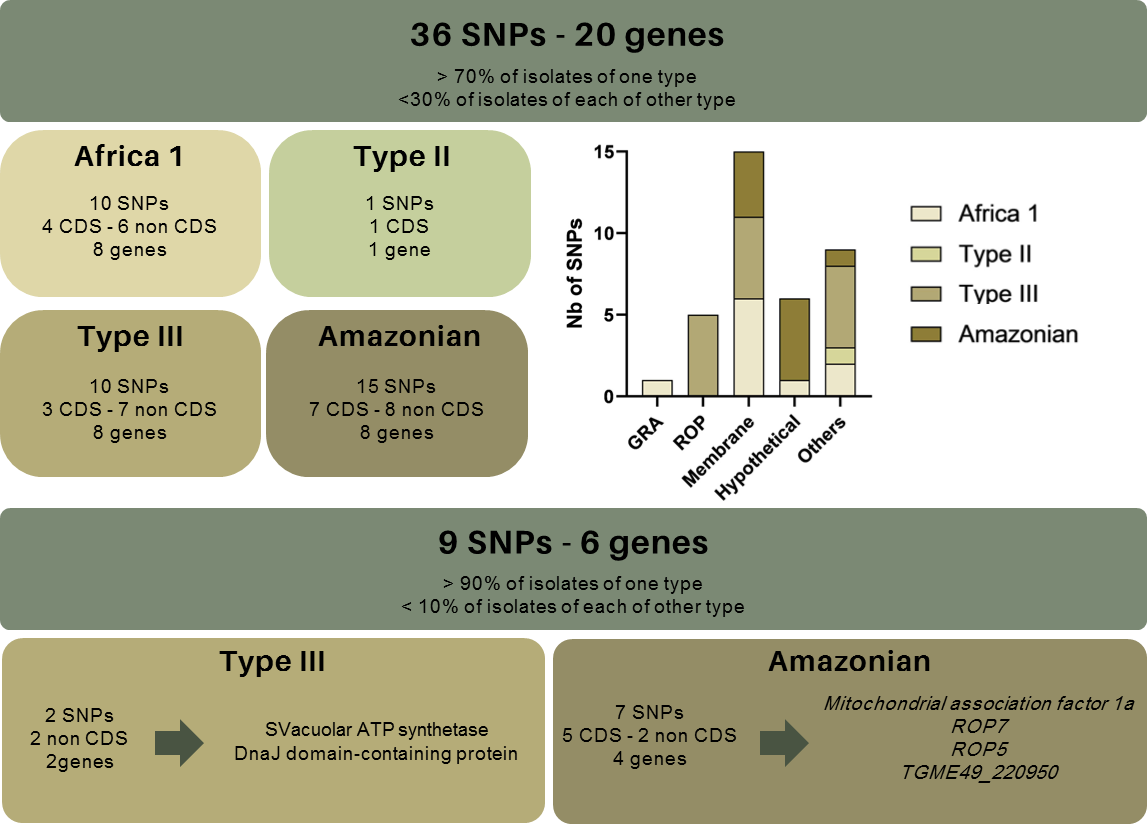
Distribution of SNPs according to the genotype of isolates. SNPs in italic indicate those associated with less than 30% of mutated isolates among the five minority types (Caribbean, Africa 3, Africa 4, Type I, Type 12). The distribution is based on the proportion of mutated SNPs for each group of isolates.

We then selected SNPs associated with mutation in 70% of isolates of one type and less than 30% of isolates of each of the other types. A total of 36 SNPs were retained, of which 10 were predominantly present in Africa 1 isolates, one in Type II, 10 in Type III, and 15 in the Amazonian type (Fig. 6). Of these 36 SNPs, six are located on genes encoding apicomplexan proteins (GRA65, ROP7, ROP5, and TGME49_308093), 15 on membrane proteins, six on hypothetical proteins, and nine on other proteins (Fig. 6). The detailed localization and nature of these 36 SNPs are presented in Table 2.

**TABLE 2:** Representative variants of the Africa 1, Type II, Type III, and Amazonian type^*a*^

Mainly represented type	Effect of the variant	Accession	Protein description	Position of mutation	Africa 1	II	III	Amazonian	Caribbean	Africa 3	Africa 4	I	12
Africa 1	3' UTR	TGME49_212310	Vacuolar ATP synthetase	c.*847T > C	**73%**	0%	5%	0%	14%	0%	0%	0%	0%
	3' UTR	TGME49_220950	Hypothetical protein	c.*1855G > T	**80%**	0%	0%	0%	0%	0%	0%	33%	0%
	Upstream	TGME49_226068	DnaJ domain-containing protein	c.-4919T > C	**73%**	0%	5%	13%	14%	0%	0%	67%	0%
	3' UTR	TGME49_233110	IMP dehydrogenase	c.*96G > C	**80%**	0%	0%	0%	**71%**	**100%**	0%	67%	0%
	3' UTR	TGME49_241240	Dense granule protein GRA65	c.*659T > C	**80%**	0%	0%	0%	**86%**	**100%**	0%	67%	0%
	Missense	TGME49_258870	Cyst wall protein CST7	c.1034A > G	**80%**	0%	0%	20%	29%	**100%**	0%	0%	0%
	Five prime	TGME49_266990	Beta-COP	c.-543A > G	**73%**	0%	0%	0%	**71%**	**100%**	0%	67%	0%
	Missense	TGME49_279100	Mitochondrial association factor 1a	c.1249G > A	**80%**	0%	0%	0%	0%	0%	0%	67%	0%
	Missense	TGME49_279100	Mitochondrial association factor 1a	c.664A > G	**73%**	0%	0%	0%	0%	0%	0%	67%	0%
	Missense	TGME49_279100	Mitochondrial association factor 1a	c.773G > A	**73%**	0%	0%	0%	0%	25%	0%	67%	0%
II	Missense	TGME49_321700	Kinase-like domain-containing protein	c.768C > A	13%	**83%**	0%	13%	0%	**100%**	0%	33%	33%
III	Missense	TGME49_204050	Subtilisin SUB1	c.1798C > T	7%	0%	**95%**	13%	29%	**100%**	**100%**	0%	0%
	Missense	TGME49_204050	Subtilisin SUB1	c.1805C > G	0%	0%	**100%**	13%	14%	**100%**	33%	0%	0%
	3' UTR	TGME49_212310	Vacuolar ATP synthetase	c.*485A > G	0%	0%	**95%**	0%	**100%**	0%	0%	0%	0%
	Upstream	TGME49_226068	DnaJ domain-containing protein	c.-2084G > A	0%	0%	**100%**	0%	**71%**	0%	0%	0%	0%
	Upstream	TGME49_244100	SnoRNA binding domain	c.-4042A > C	20%	2%	**79%**	27%	14%	25%	0%	0%	0%
	Upstream	TGME49_244100	SnoRNA binding domain	c.-4072A > C	0%	2%	**74%**	7%	0%	0%	0%	33%	33%
	Missense	TGME49_279100	Mitochondrial association factor 1a	c.190C > G	7%	2%	**89%**	7%	**86%**	**100%**	0%	33%	0%
	3' UTR	TGME49_288360	Tryptophanyl-tRNA synthetase (TrpRS2)	c.*345A > G	0%	0%	**79%**	7%	57%	0%	0%	0%	0%
	Downstream	TGME49_292120	MORN2 protein	c.*3521T > A	0%	0%	**79%**	7%	**100%**	0%	0%	0%	0%
	Upstream	TGME49_320490	Phospholipase D	c.-4194A > G	7%	0%	**74%**	27%	57%	**75%**	0%	0%	0%
Amazonian	3' UTR	TGME49_212310	Vacuolar ATP synthetase	c.*20T > A	0%	0%	0%	**73%**	0%	0%	0%	0%	0%
	Missense	TGME49_220950	Hypothetical protein	c.251T > A	0%	0%	0%	**73%**	0%	25%	0%	0%	0%
	3' UTR	TGME49_220950	Hypothetical protein	c.*655G > A	7%	0%	0%	**73%**	0%	0%	0%	0%	0%
	3' UTR	TGME49_220950	Hypothetical protein	c.*2051G > A	0%	2%	0%	**80%**	0%	0%	0%	0%	0%
	3' UTR	TGME49_220950	Hypothetical protein	c.*2243G > A	0%	0%	0%	**100%**	0%	0%	0%	0%	0%
	3' UTR	TGME49_220950	Hypothetical protein	c.*2261C > T	0%	0%	0%	**100%**	0%	0%	0%	0%	0%
	3' UTR	TGME49_251170	KRUF family protein	c.*3964G > A	0%	0%	0%	**73%**	0%	0%	0%	0%	0%
	Missense	TGME49_279100	Mitochondrial association factor 1a	c.758G > A	7%	2%	0%	**100%**	0%	0%	0%	67%	33%
	Missense	TGME49_279100	Mitochondrial association factor 1a	c.1043T > C	0%	0%	0%	**93%**	0%	0%	0%	0%	0%
	Missense	TGME49_295110	Rhoptry protein ROP7 (ROP7)	c.1040A > G	0%	0%	0%	**100%**	0%	0%	0%	33%	67%
	Missense	TGME49_295110	Rhoptry protein ROP7 (ROP7)	c.922A > G	0%	0%	0%	**73%**	0%	0%	33%	0%	0%
	Missense	TGME49_295110	Rhoptry protein ROP7 (ROP7)	c.863G > A	0%	0%	0%	**93%**	0%	0%	0%	33%	33%
	3' UTR	TGME49_301440	Calcium-dependent protein kinase CDPK1	c.*296A > G	0%	0%	0%	**73%**	0%	0%	0%	0%	0%
	Missense	TGME49_308090	Rhoptry protein ROP5 (ROP5)	c.1605C > G	0%	0%	0%	**93%**	0%	0%	0%	0%	0%
	3' UTR	TGME49_308093	Rhoptry kinase family protein	c.*658C > G	0%	0%	5%	**80%**	0%	0%	**100%**	0%	0%
Africa 1 + III	Missense	TGME49_308090	Rhoptry protein ROP5 (ROP5)	c.1483G > C	**80%**	2%	**100%**	7%	**100%**	**100%**	0%	67%	0%
	Missense	TGME49_308090	Rhoptry protein ROP5 (ROP5)	c.1378G > T	**80%**	2%	**100%**	0%	29%	**100%**	0%	0%	0%
	5' UTR	TGME49_235490	IMC sutures component ISC1	c.-82A > C	**73%**	0%	**79%**	0%	**86%**	0%	0%	0%	0%
Africa 1 + Amazonian	Missense	TGME49_279100	Mitochondrial association factor 1a	c.41C > T	**80%**	2%	0%	**100%**	0%	0%	0%	67%	0%
	Missense	TGME49_279100	Mitochondrial association factor 1a	c.428G > A	**80%**	0%	0%	**100%**	0%	0%	0%	67%	0%
	3′ UTR	TGME49_279100	Mitochondrial association factor 1a	c.*397A > G	**73%**	0%	0%	**87%**	0%	0%	0%	67%	0%
	3′ UTR	TGME49_279100	Mitochondrial association factor 1a	c.*405G > A	**73%**	0%	0%	**87%**	0%	0%	0%	67%	0%
Africa 1 + III + Amazonian	Missense	TGME49_215775	Rhoptry protein ROP8 (ROP8)	c.1612C > T	**80%**	0%	**100%**	**93%**	**100%**	0%	33%	67%	0%
	Missense	TGME49_308090	Rhoptry protein ROP5 (ROP5)	c.1307A > G	**73%**	2%	**100%**	**93%**	**100%**	**100%**	33%	67%	0%

^*a*^ Proportion of isolates mutated associated to the different variants for each type. c: variant present on the CDS; c*: variant downstream of the last exon of the CDS; c-: variant upstream of the gene transcription site. Bold: present in more than 70% of the isolates of one type.

Their distribution was also analyzed on the sequences of the 20 additional isolates corresponding to the five minority types: Caribbean (*n* = 7), Africa 3 (*n* = 4), Africa 4 (*n* = 3), Type I (*n* = 3), and Type 12 (*n* = 3). For 16/36 SNPs, the mutations are present in less than 30% of isolates of each of the five types: two SNPs are predominantly present in Africa 1, two in Type III, and 12 in Amazonian (Table 2). Of these, only seven are predominantly found in the Amazonian isolates: one on ROP5, three on ROP7, one on the hypothetical protein TGME49_220950, and two on mitochondrial association factor 1a encoding genes (Fig. 6).

Additionally, four mutations on mitochondrial association factor 1a are associated with over 70% of Africa 1 and Amazonian isolates, while two mutations on ROP5 and one on membrane protein TGME49_235490 are associated with over 70% of Africa 1 and III isolates. Finally, one mutation on ROP8 and one on ROP5 are associated with over 70% of Africa 1, III, and Amazonian isolates (Table 2).

Of the 36 SNPs identified so far, nine are associated with mutations in 90% of isolates of one type and less than 10% of isolates of each of the other types. Two SNPs are predominantly present in Type III isolates: one on the vacuolar ATP synthetase and one on the gene DnaJ domain-containing protein encoding genes. In addition, seven SNPs are mostly present in the Amazonian isolates: one on the ROP5 gene, two on the ROP7 gene, two on the gene encoding the hypothetical protein TGME49_220950, and two on the mitochondrial association factor 1a gene. Of these nine SNPs, five are present in less than 30% of the isolates belonging to the other five types (Caribbean, Africa 3 and 4, and I and 12) (Fig. 6).

## DISCUSSION

Previous studies have examined the *T. gondii* proteome and identified over 10,000 proteins, including for the Me49 strain, with variable profiles depending on the stage of the parasite (34). The roles of some of *T. gondii* proteins in infection are gradually being elucidated, yet the antigenicity of these proteins is still poorly described. Indeed, the antigenicity of only approximately 50 proteins has been described, thanks to studies evaluating their potential in vaccine immunity and to proteomic analyses. These include approximately 15 apicomplexan proteins, such as GRA4-5-6-7, ROP12-16, MIC3-8, and SAG1 and 2 (17–26, 29). The objective of this study was to identify and characterize a wide panel of proteins and protein complexes that are recognized by anti-*T*. *gondii* Ig. To this end, mice were infected with three distinct *T. gondii* type strains to produce murine sera. Sera exhibited high titers of anti-*T*. *gondii* Ab and high concentrations of total IgG, confirming the chronic phase of infection and the hyper-immunization of the mice. To avoid individual responses and increase the panel of antigenic proteins detected, sera from each mouse infection were pooled. The response to murine infection of three types of strain was analyzed: a virulent Africa 1 type (FOU, Type I-like), a non-virulent Type II (Me49), and an intermediate virulence Type III (VEG). The use of only three types of strain may restrict the panel of proteins detected due to the potential variation in protein expression profiles according to strain type. Moreover, the use of tachyzoite lysate may also limit the panel of antigenic proteins detected due to the potential variation in protein profile between the different stages of the parasite (34). Despite these limitations, Co-IP coupled to MS detection allowed us to identify for the first time a large panel of 727 antigenic *T. gondii* proteins recognized by murine anti-*T*. *gondii* Ig. These included 121 apicomplexan proteins, as well as a number of hypothetical proteins (*n* = 72). To our knowledge, 684 proteins, including 83 apicomplexan proteins, are confirmed as antigenic for the first time here. Although this large panel of proteins is derived from murine infections, it could be the source of the identification of interesting new targets to be investigated as part of vaccine or serotyping studies.

Two types of Co-IP reactions were conducted: three homologous and six heterologous reactions. The objective was to identify antigenic proteins recognized by murine Ig according to the type of the infecting strain. A third of these proteins were identified exclusively in homologous Co-IP reactions. This could indicate that they are recognized by Ig anti-*T*. *gondii* specific to the infecting strain. However, it is also possible that these proteins are present in heterologous reactions but are below the detection limit. Indeed, the quality of their identification by LC–MS/MS is low, which may suggest low concentrations of these proteins in the reactions. The remaining two-thirds of the proteins were detected in more than one of the six heterologous reactions. This may indicate that they are recognized by anti-*T*. *gondii* Ig, regardless of the type of infecting strain. It is noteworthy that these proteins encompass the majority of those for which peptides have been described as type-specific in the development of serotyping tests. The tachyzoite lysates used in this study were obtained through chemical lysis, which could potentially result in a loss of type specificity for these peptides. This could explain the observation of some of the protein previously described as type-specific in the heterologous reaction. Consequently, the results of homologous and heterologous reactions do not allow the determination of antigenic protein specificity according to the genotype.

Subsequently, an *in silico* polymorphism analysis of the 727 proteins was conducted using NGS data from 117 T. *gondii* isolates previously typed by MLST and whose genomes had been fully sequenced (1, 6). This large panel allows a substantial representation of four main strain types (i.e., African 1, II, III and Amazonian) and a lower representation of a few other types (i.e., Caribbean, Africa 3, Africa 4, Type I, and Type 12). The strains used for genetic analysis are mostly isolated from animals. It would have been interesting to have more strains isolated from human infections in order to determine the polymorphism of the strains circulating in humans. Nevertheless, the use of these strains is interesting, as the NGS genetic data from some of these isolates have been used to determine genetic classifications (6).

To our knowledge, our polymorphism study is the first to use genetic data from NGS sequencing. The use of this kind of data is interesting in the context of polymorphism studies, as NGS sequencing permits the assessment of the quality of the SNPs identified, thereby circumventing the biases associated with point mutations by retaining only those that are most accurately represented (33, 35). Furthermore, it is possible to ascertain the number of haplotypes present in a sequence and determine whether a single haplotype is predominant. In order to ensure that no potentially interesting mutations were overlooked, low-stringency quality criteria were applied to select SNPs, resulting in the exclusion of only 18% of those detected by variant calling analysis. Only those SNPs present in CDS or described regulatory regions were included. A total of 31% of the SNPs identified through variant calling analysis were subjected to further investigation. These SNPs are distributed across 704 of the 727 genes studied, and only 17% of them encode apicomplexan proteins. This finding corroborates the value of extending antigenic protein analyses to proteins other than apicomplexan, particularly in the context of serotyping work.

The study of the various SNPs revealed that the proportion of mutated isolates per type is exceedingly low. For approximately 98% of the SNPs, less than 30% of the isolates of each type are mutated. The majority of observed mutations are, therefore, very sporadic, and isolates are highly homogeneous within themselves, particularly within Type II, which is also the most represented in our study. The Caribbean types, Africa 3 and 4, and I and 12 exhibited greater heterogeneity between isolates.

A total of 36 mutations were present in over 70% of isolates of the same type and less than 30% of the other three types. Consequently, these SNPs may be associated with a type-dependent polymorphism: 10 for Africa 1, one for Type II, 10 for Type III, and 15 for Amazonian. Of the mutations of interest, 15 are located in CDS regions. Furthermore, only six of the 36 mutations were found in apicomplexan genes. One was on GRA65, one on ROP5, three on ROP7, and one on the rhoptry gene encoding TGME49_308090. This suggests that the study of other proteins could be of interest in linking a polymorphism to a genotype. It is noteworthy that 16 of the 36 SNPs are also associated with a low proportion of mutated isolates on the five other types, Caribbean, Africa 3 and 4, and I and 12. Of the 36 mutations identified, two are strongly associated with type III (non-CDS) and 7 (5 CDS/2 non-CDS) with the Amazonian type. In fact, these mutations are present in more than 90% of isolates of type III or Amazonian and in less than 10% of isolates of other types. Furthermore, five of the SNPs associated with the Amazonian type are weakly present in the five minor types: one on the ROP5 gene, one on the ROP7 gene, two on the gene encoding the hypothetical protein TGME49_220950, and one on the mitochondrial association factor 1a gene. Given the sporadic nature of the mutations observed in the previous types (Africa 1, II, III, and Amazonian) and the limited number of isolates analyzed for these five other types (from three to seven), it is challenging to ascertain the presence or absence of these mutations within these types. It would be necessary to include a larger number of isolates to best represent the five genotypes and account for individual variability and potential sequencing biases to confirm the interest of these mutations. Furthermore, given the low variability and the *a priori* heterogeneous distribution of the mutations observed here, it would be interesting to include data from other genotypes in order to confirm the specificity of the mutations observed.

In total, the *in silico* polymorphism analysis identified 36 SNPs for which the mutation could be linked to a specific type. Of these, nine were found to be more strongly associated with two types: III and Amazonian. Among these, five CDS mutations associated with the Amazonian type are present in less than 30% of isolates of all other types: one on ROP5, two on ROP7, one on the hypothetical protein TGME49_220950, and two on mitochondrial association factor 1a encoding genes. Only 15 of the 36 SNPs are present in coding sequence regions, but *in silico* analyses did not reveal any epitopes linked to these mutations (data not shown). While it is challenging to ascertain the specificity of these mutations with regard to type solely on the basis of genetic data, it may be worthwhile to develop peptides from these mutations and evaluate their specificity, both individually and in combination, with the aim of enhancing their discriminatory power, for example, against sera of Guyanese patients. The remaining mutations are located in regulatory regions and could result in splicing variations, overexpression, or underexpression of target proteins, which may be dependent on the type. However, without experimental data suggesting that these SNPs actually alter antigenic protein levels, this argument remains hypothetical. Therefore, it would be prudent not to overinterpret these findings without further experimental confirmation. Future studies, including functional analyses, will be necessary to confirm the impact of these mutations on protein expression and their potential role in genotype specificity for serotyping. Additionally, it would be valuable to assess the potential impact of polymorphisms in multicopy genes, such as *ROP5* and *ROP8*, where mutations may vary in their relevance and expression across different types, to better understand their possible role in type differentiation.

### Conclusion

Our study represents a significant advancement in the field of antigenic protein identification, as it is the first to use an *in vivo* approach. This approach has enabled the discovery of a large panel of antigenic proteins, the majority of which have not been previously described as antigenic. These proteins offer a valuable resource for vaccine and serotyping research. To the best of our knowledge, our *in silico* polymorphism study is the first to be based on NGS-type genetic data on a large number of isolates for four types of varying virulence. Among the genes encoding the antigenic proteins identified, the observed polymorphism is low; however, it enables the identification of mutations that could be type-specific. Nevertheless, further studies are necessary to confirm the specificity and clinical usefulness of these mutations.

## Data Availability

Data are available at 10.6084/m9.figshare.27118695.

## SUPPLEMENTAL MATERIAL

Tables S1 and S2 spectrum.02040-24-s0001.docx Table S1: Characterization of murine sera. Table S2: *Toxoplasma gondii* strain description used for the polymorphism study.

ASM does not own the copyrights to Supplemental Material that may be linked to, or accessed through, an article. The authors have granted ASM a non-exclusive, world-wide license to publish the Supplemental Material files. Please contact the corresponding author directly for reuse.
