# Supplementary material for: Identification of Toxoplasma gondii antigenic proteins using an in vivo approach and in silico investigation of their polymorphism
Source: Microbiol Spectr. 2025 Mar 26;13(5):e02040-24. doi: 10.1128/spectrum.02040-24 (PMC12054087; doi:10.1128/spectrum.02040-24)
Supplement: Tables S1 and S2 — Table S1: Characterization of murine sera. Table S2: Toxoplasma gondii strain description used for the polymorphism study. [file spectrum.02040-24-s0001.docx]

| **Strain name** | **Infection number** | **HAI  Ig titer** | **IgG1  (ng/mL)** | **IgG2a  (ng/mL)** | **IgG2b  (ng/mL)** | **IgG3  (ng/mL)** | **IgM  (ng/mL)** |
| --- | --- | --- | --- | --- | --- | --- | --- |
| FOU | 1 | 1/320 | 2778 | 1331486 | 21089 | Sup to 1383136 | 3283 |
| FOU | 2 | 1/160 | 10461 | 1331486 | 610010 | Sup to 1383136 | 1591 |
| FOU | 3 | 1/80 | 6 | 4999 | 8841 | Sup to 1383136 | 709 |
| Me49 | 1 | 1/2560 | 16224 | 1331486 | 18013 | Sup to 1383136 | 3715 |
| Me49 | 2 | 1/2560 | 1321 | 18952 | 1071 | Sup to 1383136 | 659 |
| Me49 | 3 | 1/2560 | 3948 | 53584 | 11751 | Sup to 1383136 | 877 |
| VEG | 1 | 1/2560 | 4 | 2880 | 2352 | Sup to 1383136 | 773 |
| VEG | 2 | 1/2560 | 11177 | 1331486 | 4177 | Sup to 1383136 | 1186 |
| VEG | 3 | 1/2560 | 65 | 7062 | 3247 | 17111 | 121 |

**Table S1 : Characterization of murine sera.** HAI (Hemagglutination) for semi-quantitative determination of serum antibodies to *T. gondii* was performed using the ELI.H.A® TOXO kit (Elitech). Total serum IgG was determined using the Procartaplex Mouse Antibody Isotyping Panel 7-plexTM kit (Invitrogen) according to the manufacturer's recommendations. Each infection was performed on triplicate (3 mice). Sup : Superior.

**Table S2:** *Toxoplasma gondii* strains description used for polymorphism study

| Strain name | Alternative names | WGS sequencing | accession number | Multilocus Microsatellite classification |
| --- | --- | --- | --- | --- |
| BENIN01 | P637S1AJ14 | Galal et al., 2022 | ERS13421591 | Africa 1 |
| BENIN02 | P19S1AJ6 | Galal et al., 2022 | ERS13421592 | Africa 1 |
| BENIN03 | P193S1CJ28 | Galal et al., 2022 | ERS13421595 | Type III |
| BENIN04 | P82S1AJ6 | Galal et al., 2022 | ERS13421593 | Africa 1 |
| BENIN05 | P63S1AJ6 | Galal et al., 2022 | ERS13421594 | Africa 1 |
| BOF | BOF ; BE-BOF | Lorenzi et al., 2016 | SRX099774 | Africa 1 |
| CAMEROON01 | BCB007-MOU | Galal et al., 2022 | ERS13421597 | Africa 1 |
| DROC01 | PSP026-2005-MUP | Galal et al., 2022 | ERS13421598 | Type III |
| FOU | FOU ; BRE-FOU | Lorenzi et al., 2016 | SRX038725 | Africa 1 |
| FRANCE01 | FR-Gal dom-006 | Galal et al., 2022 | ERS13421599 | Type II |
| FRANCE02 | FR-Gal dom-007 | Galal et al., 2022 | ERS13421600 | Type II |
| FRANCE03 | FR-Vul vul-017 | Galal et al., 2022 | ERS13421601 | Type II |
| FRANCE04 | FR-Sus scr-063 | Galal et al., 2022 | ERS13421602 | Type II |
| FRANCE05 | 190201Bbut002 | Galal et al., 2022 | ERS13421603 | Type II |
| FRANCE06 | 190312Rnor01 | Galal et al., 2022 | ERS13421604 | Type II |
| FRANCE07 | 190419Bbut001 | Galal et al., 2022 | ERS13421605 | Type II |
| FRANCE08 | FR-Cap cap-009 | Galal et al., 2022 | ERS13421606 | Type II |
| FRANCE09 | FR-Cap cap-012 | Galal et al., 2022 | ERS13421607 | Type II |
| FRANCE10 | FR-Ovi ari-061 | Galal et al., 2022 | ERS13421608 | Type II |
| FRANCE11 | FR-Sus scr-015 | Galal et al., 2022 | ERS13421609 | Type II |
| FRANCE12 | FR-Ovi ari-011 | Galal et al., 2022 | ERS13421610 | Type II |
| FRANCE13 | FR-Bos tau-001 | Galal et al., 2022 | ERS13421611 | Type II |
| FRANCE14 | FR-Vul vul-031 | Galal et al., 2022 | ERS13421612 | Type II |
| FRANCE15 | FR-Sus scr-027 | Galal et al., 2022 | ERS13421613 | Type II |
| FRANCE16 | FR-Sus scr-034 | Galal et al., 2022 | ERS13421614 | Type II |
| FRANCE17 | FR-Sus scr-035 | Galal et al., 2022 | ERS13421615 | Type II |
| FRANCE18 | FR-Sus scr-039 | Galal et al., 2022 | ERS13421616 | Type II |
| FRANCE19 | FR-Sus scr-047 | Galal et al., 2022 | ERS13421617 | Type II |
| FRANCE20 | FR-Sus-scr-062 | Galal et al., 2022 | ERS13421622 | Type III |
| FRANCE21 | FR-Vul vul-056 | Galal et al., 2022 | ERS13421618 | Type II |
| FRANCE22 | FR-Vul vul-068 | Galal et al., 2022 | ERS13421619 | Type II |
| FRANCE23 | CAE013-PIR | Galal et al., 2022 | ERS13421620 | Type II |
| FRANCE24 | CCH058-2009-GRO | Galal et al., 2022 | ERS13421621 | Type II |
| FRENCHGUIANA01 | GUY-CAN-FAM-009 | Galal et al., 2022 | ERS13421629 | Caribbean 1 |
| FRENCHGUIANA02 | GUY-CAN-FAM-018 | Galal et al., 2022 | ERS13421630 | Caribbean 2 |
| FRENCHGUIANA05 | GUY-CAN-FAM-008 | Galal et al., 2022 | ERS13421631 | Type III |
| FRENCHGUIANA06 | GUY-FEL-CAT-009 | Galal et al., 2022 | ERS13421632 | Type III |
| FRENCHGUIANA08 | GUY-GAL-VIT-001 | Galal et al., 2022 | ERS13421623 | Amazonian |
| FRENCHGUIANA09 | GUY-CAN-FAM-007 | Galal et al., 2022 | ERS13421624 | Amazonian |
| FRENCHGUIANA11 | GUY013-2004-LAB | Galal et al., 2022 | ERS13421625 | Amazonian |
| FRENCHGUIANA12 | VAND ; GUY-VAND | Lorenzi et al., 2016 | SRX038726 | Amazonian |
| FRENCHGUIANA13 | GUY066-MON | Galal et al., 2022 | ERS13421626 | Amazonian |
| FRENCHGUIANA14 | GUY071-GUY | Galal et al., 2022 | ERS13421627 | Amazonian |
| FRENCHGUIANA15 | GUYS006-BAY | Galal et al., 2022 | ERS13421628 | Amazonian |
| FRENCHGUIANA16 | GUY-DOS | Lorenzi et al., 2016 | SRX099782 | Amazonian |
| FRENCHGUIANA17 | GUY-KOE | Lorenzi et al., 2016 | SRX099796 | Amazonian |
| FRENCHGUIANA18 | GUY-MAT | Lorenzi et al., 2016 | SRX099783 | Amazonian |
| FRENCHGUIANA19 | GUY-2003-MEL | Lorenzi et al., 2016 | SRX160131 | Amazonian |
| FRENCHGUIANA20 | GUY-2004-ABE | Lorenzi et al., 2016 | SRX160132 | Amazonian |
| FRENCHGUIANA21 | GUY009-AKO | Lorenzi et al., 2016 | SRX171132 | Amazonian |
| FRENCHGUIANA22 | GUY 021 - TOJ | Lorenzi et al., 2016 | SRX160041 | Amazonian |
| FRENCHGUIANA24 | RUB ; GUY-RUB | Lorenzi et al., 2016 | SRX099773 | Amazonian |
| GABON01 | GAB1-CAP-AEG007 | Galal et al., 2022 | ERS13421637 | Type III |
| GABON03 | GAB1-FEL-CAT001 | Galal et al., 2022 | ERS13421638 | Type III |
| GABON05 | GAB3-GAL-DOM013 | Galal et al., 2022 | ERS13421639 | Type III |
| GABON07 | GAB7-GAL-DOM007 | Galal et al., 2022 | ERS13421640 | Type III |
| GABON08 | GAB3-2007-GAL-DOM2 | Lorenzi et al., 2016 | SRX160123 | Africa 1 |
| GABON09 | GAB5-2007-GAL-DOM1 | Lorenzi et al., 2016 | SRX160069 | Africa 1 |
| GABON10 | GAB1-2007-GAL-DOM10 | Lorenzi et al., 2016 | SRX159841 | Africa 3 |
| GABON11 | GAB2-2007-GAL-DOM2 | Lorenzi et al., 2016 | SRX156037 | Africa 3 |
| GABON12 | GAB3-2007-GAL-DOM9 | Lorenzi et al., 2016 | SRX160125 | Africa 3 |
| GABON13 | GAB5-2007-GAL-DOM6 | Lorenzi et al., 2016 | SRX159842 | Africa 3 |
| GUADELOUPE02 | PAP002-2010-GOM | Galal et al., 2022 | ERS13421644 | Caribbean 2 |
| GUYANA01 | TgCkGy2 | Lorenzi et al., 2016 | SRX099785 | Caribbean 2 |
| MARTINIQUE01 | FDF001-ANG | Galal et al., 2022 | ERS13421649 | Type II |
| MARTINIQUE02 | FDF010-ANS | Galal et al., 2022 | ERS13421647 | Caribbean 2 |
| MARTINIQUE03 | FDF012-MAN | Galal et al., 2022 | ERS13421648 | Caribbean 3 |
| MARTINIQUE04 | FDF012-DEA | Galal et al., 2022 | ERS13421646 | Caribbean 1 |
| PORTUGAL01 | PT-2004-SUS SCR001 | Galal et al., 2022 | ERS13421651 | Type II |
| PORTUGAL02 | PT-SUS SCR002 | Galal et al., 2022 | ERS13421657 | Type III |
| PORTUGAL03 | PT-2005-SUS SCR004 | Galal et al., 2022 | ERS13421652 | Type II |
| PORTUGAL04 | PT-SUS SCR005 | Galal et al., 2022 | ERS13421658 | Type III |
| PORTUGAL05 | PT-2005-SUS SCR006 | Galal et al., 2022 | ERS13421653 | Type II |
| PORTUGAL06 | PT-2005-SUS SCR007 | Galal et al., 2022 | ERS13421654 | Type II |
| PORTUGAL07 | PT-2005-SUS SCR 11 | Galal et al., 2022 | ERS13421655 | Type II |
| PORTUGAL08 | PT-2005-SUS SCR013 | Galal et al., 2022 | ERS13421656 | Type II |
| PORTUGAL09 | PT-SUS SCR014 | Galal et al., 2022 | ERS13421659 | Type III |
| PORTUGAL10 | PT-B1 | Galal et al., 2022 | ERS13421660 | Type I |
| SENEGAL01 | 160817Gdom21 | Galal et al., 2022 | ERS13421668 | Type II |
| SENEGAL02 | 160823Gdom12 | Galal et al., 2022 | ERS13421669 | Type II |
| SENEGAL03 | 180218Gdom96 | Galal et al., 2022 | ERS13421670 | Type II |
| SENEGAL04 | 160504Fcat01 | Galal et al., 2022 | ERS13421665 | Africa 4 |
| SENEGAL05 | 160817Gdom23 | Galal et al., 2022 | ERS13421661 | Africa 1 |
| SENEGAL06 | 160730Gdom13 | Galal et al., 2022 | ERS13421671 | Type II |
| SENEGAL07 | 160421Gdom10 | Galal et al., 2022 | ERS13421672 | Type II |
| SENEGAL08 | 160416Gdom13 | Galal et al., 2022 | ERS13421673 | Type II |
| SENEGAL09 | 160421Gdom18 | Galal et al., 2022 | ERS13421682 | Type III |
| SENEGAL10 | 160428Gdom06 | Galal et al., 2022 | ERS13421662 | Africa 1 |
| SENEGAL12 | 160518Gdom05 | Galal et al., 2022 | ERS13421674 | Type II |
| SENEGAL13 | 160529Gdom26 | Galal et al., 2022 | ERS13421675 | Type II |
| SENEGAL14 | 160530Cmos12 | Galal et al., 2022 | ERS13421666 | Africa 4 |
| SENEGAL15 | 160614Gdom21 | Galal et al., 2022 | ERS13421683 | Type III |
| SENEGAL16 | 160615Gdom02 | Galal et al., 2022 | ERS13421676 | Type II |
| SENEGAL17 | 160622Gdom02 | Galal et al., 2022 | ERS13421677 | Type II |
| SENEGAL18 | 160622Gdom38 | Galal et al., 2022 | ERS13421678 | Type II |
| SENEGAL19 | 160623Gdom08 | Galal et al., 2022 | ERS13421679 | Type II |
| SENEGAL20 | 160623Gdom19 | Galal et al., 2022 | ERS13421684 | Type III |
| SENEGAL21 | 160625Gdom11 | Galal et al., 2022 | ERS13421680 | Type II |
| SENEGAL22 | 160628Gdom17 | Galal et al., 2022 | ERS13421663 | Africa 1 |
| SENEGAL23 | 160628Gdom28 | Galal et al., 2022 | ERS13421667 | Africa 4 |
| SENEGAL24 | 160628Gdom35 | Galal et al., 2022 | ERS13421681 | Type II |
| SENEGAL25 | 160816Gdom37 | Galal et al., 2022 | ERS13421664 | Africa 1 |
| SENEGAL26 | 160823Gdom04 | Galal et al., 2022 | ERS13421685 | Type III |
| SPAIN01 | 170329Lmic01ES | Galal et al., 2022 | ERS13421688 | Type II |
| TUNISIA01 | TUN-Oviari-066 | Galal et al., 2022 | ERS13421689 | Type II |
| TUNISIA02 | TUN-Oviari-071 | Galal et al., 2022 | ERS13421691 | Type III |
| TUNISIA03 | TUN-Oviari-074 | Galal et al., 2022 | ERS13421690 | Type II |
| TURKEY01 | Ankara LS-1 | Galal et al., 2022 | ERS13421692 | Africa 1 |
| TURKEY02 | TR-EGE1 LS-2 | Galal et al., 2022 | ERS13421693 | Africa 1 |
| UK01 | NAN033-2005-PLA | Galal et al., 2022 | ERS13421694 | Type II |
| USA01 | US-CT1 | Galal et al., 2022 | ERS13421695 | Type I |
| USA03 | GT1 | Lorenzi et al., 2016 | SRX156314 | Type I |
| USA04 | M7741 | Lorenzi et al., 2016 | SRX159890 | Type III |
| USA10 | VEG | Lorenzi et al., 2016 | SRX156300 | Type III |
| USA11 | B41 | Lorenzi et al., 2016 | SRX099774 | Type 12 |
| USA12 | ARI | Lorenzi et al., 2016 | SRX099777 | Type 12 |
| USA13 | RAY | Lorenzi et al., 2016 | SRX099793 | Type 12 |
